# Systemic delays in the initiation of antiretroviral therapy during pregnancy do not improve outcomes of HIV-positive mothers: a cohort study

**DOI:** 10.1186/1471-2393-12-94

**Published:** 2012-09-11

**Authors:** Landon Myer, Rose Zulliger, Linda-Gail Bekker, Elaine Abrams

**Affiliations:** 1 Centre for Infectious Diseases Epidemiologic Research, School of Public Health & Family Medicine, University of Cape Town, Anzio Road, Observatory 7925, Cape Town, South Africa; 2 Department of Health, Behavior & Society, Johns Hopkins Bloomberg School of Public Health, 615 North Wolfe Street, Baltimore, Maryland, 21205, USA; 3 Desmond Tutu HIV Centre, Institute of Infectious Diseases & Molecular Medicine, University of Cape Town, Anzio Road, Observatory 7925, Cape Town, South Africa; 4 ICAP, Mailman School of Public Health & College of Physicians & Surgeons, Columbia University, 722 West 168th Street, New York, New York, 10032, USA

**Keywords:** Antiretroviral therapy, Pregnancy, Patient preparation, Prevention of mother-to-child transmission (PMTCT), HIV/AIDS, South Africa

## Abstract

**Background:**

Antiretroviral therapy (ART) initiation in eligible HIV-infected pregnant women is an important intervention to promote maternal and child health. Increasing the duration of ART received before delivery plays a major role in preventing vertical HIV transmission, but pregnant women across Africa experience significant delays in starting ART, partly due the perceived need to deliver ART counseling and patient education before ART initiation. We examined whether delaying ART to provide pre-ART counseling was associated with improved outcomes among HIV-infected women in Cape Town, South Africa.

**Methods:**

We undertook a retrospective cohort study of 490 HIV-infected pregnant women referred to initiate treatment at an urban ART clinic. At this clinic all patients including pregnant women are screened by a clinician and then undergo three sessions of counseling and patient education prior to starting treatment, commonly introducing delays of 2–4 weeks before ART initiation. Data on viral suppression and retention in care after ART initiation were taken from routine clinic records.

**Results:**

A total of 382 women initiated ART before delivery (78%); ART initiation before delivery was associated with earlier gestational age at presentation to the ART service (p < 0.001). The median delay between screening and ART initiation was 21 days (IQR, 14–29 days). Overall, 84.7%, 79.6% and 75.0% of women who were pregnant at the time of ART initiation were retained in care at 4, 8 and 12 months after ART initiation, respectively. Among those retained, 91% were virally suppressed at each follow-up visit. However the delay from screening to ART initiation was not associated with retention in care and/or viral suppression throughout the first year on ART in unadjusted or adjusted analyses.

**Conclusions:**

A substantial proportion of eligible pregnant women referred for ART do not begin treatment before delivery in this setting. Among women who do initiate ART, delaying initiation for patient preparation is not associated with improved maternal outcomes. Given the need to maximize the duration of ART before delivery for prevention of mother-to-child HIV transmission, there is an urgent need for new strategies to help expedite ART initiation in eligible pregnant women.

## Background

There were an estimated 12 million HIV-infected women of reproductive age living in sub-Saharan Africa during 2011, and 3.3 million in South Africa (SA) alone [1]. In SA, the high national antenatal HIV seroprevalence (30%) means that a large number of pregnant women require services to prevent the mother-to-child transmission (PMTCT) of HIV infection [2]. Because most perinatal HIV transmission takes place in women with advanced HIV disease who are eligible for lifelong antiretroviral therapy (ART) [3], ART initiation in pregnancy is a critical intervention both for PMTCT and for the long-term health of mothers [4]. In this context, increasing the time on ART before delivery contributes to reductions in viraemia and decreases the risk of HIV transmission *in utero*, during labour and delivery, and postpartum when breastfeeding [5,6]. Several studies have demonstrated that a one-week increase in the duration of ART received antenatally is associated with an approximately 10% decrease in the risk of mother-to-child transmission of HIV [7-9], making rapid ART initiation in eligible pregnant women an important goal for effective PMTCT services [10].

Despite the importance of rapid ART initiation for PMTCT, across sub-Saharan Africa most pregnant women face significant delays to starting ART [11]. Eligible pregnant women are typically referred to general adult ART clinics for treatment [12]. Once at ART clinics, pregnant women enter a standardized system of assessment and patient education that can delay ART initiation by several weeks. In most settings, the systems for adult ART initiation are focused on the needs of the general adult population of non-pregnant women and men, as pregnant women comprise only a small fraction of new patients. [4,13] In the general population of non-pregnant adults, systemic delays in ART initiation are routinely used to allow time for patient education and psychosocial preparation before treatment [14], based on the idea that patient preparation before initiation may improve retention in care and treatment adherence over time [15-17], although evidence for this is lacking [18].

Systems that introduce delays in ART initiation for patient preparation before beginning treatment may have important potential benefits for some adults. Yet in the context of pregnancy, such delays can contribute to increased risk of mother-to-child transmission of HIV. Thus there is a potential risk-benefit balance to delaying ART initiation in pregnancy: delays in ART initiation may be useful to ensure adequate patient preparation (in order to improve retention and adherence on treatment) but also reduce the duration of ART received before delivery (and thus contribute to the vertical transmission of HIV by delaying viral suppression). There are few data to inform this risk-benefit assessment however. In particular, there are few studies of whether delayed ART initiation for patient preparation improves patient outcomes, including in the context of pregnancy where delaying ART initiation may contribute to HIV transmission risk. To address this, we examined the impact of systemic delays in ART initiation in eligible pregnant women on treatment outcomes in Cape Town, South Africa.

## Methods

We undertook a retrospective cohort study of HIV-infected women initiating ART in the community of Gugulethu, where the antenatal seroprevalence in 2011 was more than 25%. The ART clinic serving Gugulethu has been in operation since 2003 and more than 9000 patients have been seen within the service [19,20].

### ART service

In Gugulethu, pregnant women who are identified as ART-eligible according to immunological (CD4 cell count <200 cells/μL until 2008, <250 cells/μL from 2008 to 2010, and <350 cells/μL since 2010) and/or clinical criteria (WHO stage IV disease) at the nearby antenatal clinic are referred for ART. Pregnant women are routinely initiated on zidovudine prophylaxis at the antenatal clinic, and continue this throughout the screening period until ART initiation. At the ART clinic all patients, including pregnant women, undergo three sessions of counseling and ART education prior to starting treatment. This often introduces a systemic delay of two to four weeks into the process of ART initiation, although individual clinicians can reduce this pre-ART screening period. All patients, including women initiating ART during pregnancy, receive care free of charge and are started on a regimen of 2 nucleoside reverse transcriptase inhibitors and a non-nucleoside reverse transcriptase inhibitor. Patients are followed-up at monthly intervals for the first 4 months on ART, then 4-monthly for the first 12 months on treatment. Immunological and virologic assessments are conducted at 4, 8 and 12 months on ART.

### Data collection

We abstracted data from patient records for the 490 pregnant women who attended ART services in Gugulethu between 2003 and 2010. We included data on: demographic characteristics; the obstetric and clinical history of women as recorded in patient records; dates of clinic visits (including initial screening, ART initiation, and subsequent follow-up visits); reasons for delays in ART initiation; clinical information on staging and opportunistic infections; and results of CD4 cell counts and viral load testing. Ethical approval to abstract data and conduct this analysis was provided by the Human Research Ethics Committee of the University of Cape Town.

### Analysis

Data were analyzed using Stata Version 11.0 (Stata Corporation, College Station, USA). The principle exposure of interest was the delay in days from women’s first screening visit at the ART clinic to the date of ART initiation; this was analysed as a continuous variable and categorized into <14, 14–20, 21–34 and ≥35 days. We examined other cutpoints, including a binary schema, but this did not change the results substantively. The principle outcomes of interest were retention in care and virological suppression during the first 12 months on treatment. Loss to follow-up (ie, failure to be retained in care) was defined as having 60 days elapsed since the last scheduled visit. Viral suppression was defined as <1000 copies/mL at any visit. Data were summarized using proportions or medians with interquartile ranges (IQR). Bivariate analyses used rank-sum and Kruskall-Wallis tests (for continuous variables) and Fisher’s exact tests (for categorical variables); all statistical tests are 2-sided at alpha = 0.05. Scatterplots with linear or locally-weighted scatterplot smothing lines were used to graphically portray the association between key variables. We examined the association between delays to ART initiation and women’s subsequent retention in care and viral suppression during the first 12 months after ART initiation using log-linked regression models with robust standard errors. In the model building process, we examined a range of potential variables of interest, including calendar year and baseline demographic, clinical and laboratory measures; variables were retained in the analysis if they were the *a priori* exposures of interest (gestational age at screening and delay from screening to ART initiation), if they were independently associated with the outcome of interest, or if their inclusion changed other associations in the model appreciably. Model fit was examined using likelihood ratio testing as well as Akaike’s Information Criterion. The model results are presented as risk ratios (RR) with 95% confidence intervals (CI).

## Results

**Figure 1:**
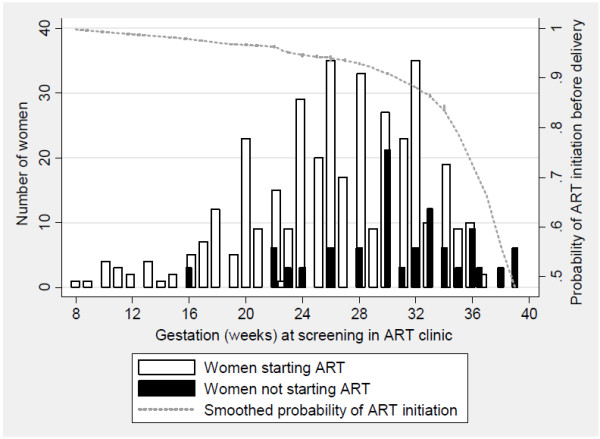
**Plot of gestation at first screening visit at antiretroviral therapy (ART) clinic (x-axis) among 408 pregnant women screened for ART, with number of women who did and did not start ART before delivery, as well as smoothed probability of ART initiation before delivery.** Smoothed probabilities are from locally weighted scatterplot smoothing (bandwidth, 0.8).

Of the 490 ART-eligible pregnant women who were seen at the Gugulethu ART clinic, the median age was 27 years (IQR, 24–31) and the median gestational age was 28 weeks (IQR, 24–32) at the time of the screening visit. The nadir CD4 cell count at screening increased from 125 cells/uL in 2004 to 205 cells/uL in 2010 (p < 0.001). A total of 108 women (22%) did not initiate ART before delivery; women who did not start treatment before delivery presented to the ART clinic at a significantly later gestational age compared to women who did initiate before delivery (median gestation age, 31 vs 27 weeks, respectively, p < 0.001; Figure 1). There was no difference in women’s age, nadir CD4 cell count or baseline viral load comparing those who were initiated during pregnancy and those who were not (p > 0.2 for all associations).

Table 1 describes the demographic, obstetric and clinic characteristics of the 382 women who started ART during pregnancy. In this group, the median nadir CD4 cell count was 142 cells/μL (IQR, 96–186) and the median log viral load was 4.4 log_10_ copies/mL (IQR, 3.7-4.8). Almost three-quarters of women presented to the ART clinic after 24 weeks’ gestation, and 13% (50) presented after 32 weeks’ gestation.

**Table 1:** Baseline description of 382 women initiating ART during pregnancy, by delay in days from screening to ART initiation

	**Not initiating ART during antenatal period**	**Total initiating ART during antenatal period**	***Delay from screening to ART initiation during antenatal period***					**p-value**
			**<14 days**	**14-20 days**	**21-27 days**	**28-34 days**	**35+ days**	
Number of patients	108	382	54	136	61	66	65	--
Median age (IQR)	26 (23–31)	27 (24–31)	29 (24–32)	27 (24–31)	28 (25–32)	27 (25–31)	27 (25–31)	0.965
Median gestation (IQR)	31 (27–34)	27 (23–31)	32 (28–34)	28 (26–32)	27 (23–30)	24 (20–28)	21 (17–25)	<0.001
<24 weeks	12 (12)	104 (27)	3 (6)	14 (10)	16 (26)	30 (45)	41 (63)	<0.001
24-28 weeks	25 (24)	134 (35)	12 (22)	57 (42)	26 (43)	21 (32)	18 (28)	
29-32 weeks	33 (31)	94 (25)	19 (35)	40 (29)	14 (23)	15 (23)	6 (9)	
>32 weeks	34 (33)	50 (13)	20 (37)	25 (18)	5 (8)	0	0	
WHO stage: I	67 (62)	206 (55)	25 (48)	83 (62)	38 (62)	34 (52)	26 (40)	0.003
II	17 (16)	69 (18)	7 (13)	29 (22)	10 (16)	13 (20)	10 (15)	
III	22 (20)	90 (24)	17 (33)	18 (13)	13 (21)	15 (23)	27 (42)	
IV	2 (2)	13 (3)	3 (6)	4 (3)	0	4 (6)	2 (3)	
Median CD4 cell count (IQR) *	120 (65–166)	142 (96–186)	139 (85–187)	143 (96–186)	153 (112–210)	133 (104–170)	134 (94–172)	0.538
CD4 <100 cells/μl	9 (39)	99 (28)	19 (36)	38 (29)	11 (19)	15 (24)	16 (29)	
Median HIV Viral load (IQR) *	4.9 (4.6-4.9)	4.3 (3.7-4.8)	4.2 (3.4-4.8)	4.2 (3.8-4.8)	4.4 (3.9-4.9)	4.5 (3.9-4.9)	4.6 (4.1-4.8)	0.211
>5 log_10_ copies/mL	3 (15)	55 (15)	9 (17)	17 (13)	8 (14)	13 (21)	8 (15)	

* For the 108 women not starting ART, CD4 data were available on 23 patients (21%) and viral load data were available on 22 patients (20%); for the 382 women starting ART, CD4 data were available on 357 patients (93%) and viral load data were available on 358 patients (94%).

The median delay between screening and ART initiation was 21 days (IQR, 14–29 days; range: 2–105 days), with 54 women (14%) starting ART less than 2 weeks after the date of screening and 111 women (29%) starting ART 28 or more days after screening. The most commonly noted reasons for delays to ART initiation of more than 28 days were patients’ late attendance or missed ART clinic visits (cited in 42% of instances, n = 87) or women’s failure to complete patient education sessions and/or a home visit (n = 65, 31%) (of 172 patients with explanations for the delay to ART initiation; more than one reason for delay was possible). A total of 19 women (11%) were delayed due to medical concerns, primarily suspicion of tuberculosis.

**Figure 2:**
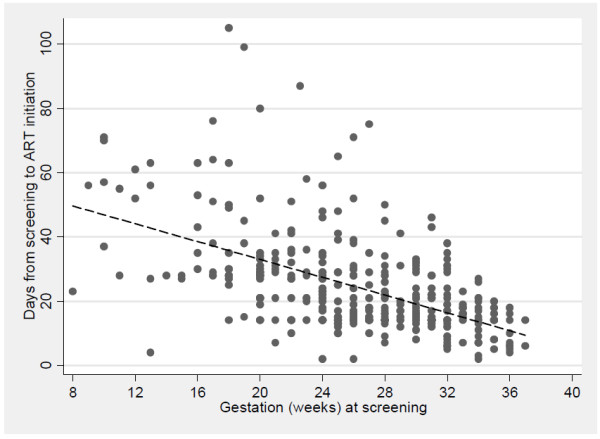
**Plot of gestation at first screening visit in ART clinic (x-axis) and delay between screening and ART initiation before delivery (y-axis), among 382 pregnant women initiating ART.** Points are individual observations, with best-fit line summarizing the overall association.

There was a strong correlation between increased gestation at first screening and decreased delay between screening and ART initiation: 40% of women screened after 32 weeks’ gestation started ART in less than 14 days, compared to 3% of women who were screened before 24 weeks’ gestation (p < 0.001; Figure 2). The median gestation at ART initiation was 30 weeks (IQR, 23–31) and the median duration of ART received before delivery was 8 weeks (IQR, 5–13 weeks). Increasing duration of ART before delivery was associated with shorter delays between screening and ART initiation, independent of gestation at first screening.

**Figure 3:**
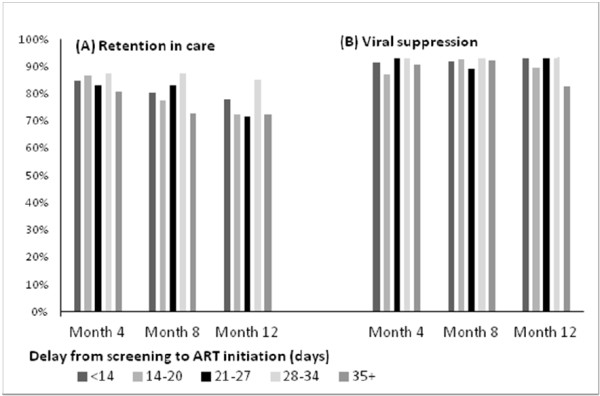
Levels of (A) retention in care and (B) viral suppression at 4, 8 and 12 months after antiretroviral therapy (ART) initiation among women who were pregnant at the time of ART initiation, according to the delay period from screening to ART initiation.

Figure 3 shows the levels of patient retention and viral suppression at months 4, 8 and 12 following ART initiation according to the delay between screening and ART initiation. Overall, 84.7%, 79.6% and 75.0% of women who were pregnant at the time of ART initiation were retained in care at 4, 8 and 12 months after ART initiation, respectively (there were 11 deaths during the first 12 months on ART, with no differences by category of delay from screening to ART initiation). Among women retained, 91% were virally suppressed to <1000 copies/mL at each of these visits.

In regression modeling (Table 2), the delay from screening to ART initiation was not associated with the combined endpoint of retention in care and viral suppression during the first year on ART in unadjusted or adjusted analyses. After adjusting for women’s age and gestational age at screening, women initiating ART 14–20, 21–27, 28–34 and more than 35 days after screening were not significantly more or less likely to be retained in care and virally suppressed, compared to women starting ART less than 14 days after screening (RR: 0.95, 0.97, 1.07 and 0.89, respectively). This finding did not change when the delay variable was categorized as a binary variable around the median of 21 days (not shown). In the final model, increasing participant age was the only factor associated with increasing probability of retention in care and viral suppression over time.

**Table 2:** Unadjusted (panel A) and adjusted (panel B) regression models predicting the combined endpoint of retention in care and viral suppression during the first 12 months on ART

	***(A) Unadjusted associations***			(B) Adjusted associations *		
	**RR**	**95% CI**	**p-value**	**RR**	**95% CI**	**p-value**
*Patient age*						
<25 years	1.0	(ref)		1.0	(ref)	
25-30 years	1.09	0.95-1.27	0.212	1.10	0.95-1.28	0.192
>30 years	1.19	1.03-1.39	0.019	1.20	1.03-1.39	0.018
*Gestation at screening*						
<24 weeks	1.0	(ref)		1.0	(ref)	
24-28 weeks	0.99	0.86-1.13	0.875	0.95	0.81-1.10	0.476
29-32 weeks	0.97	0.84-1.13	0.722	0.93	0.78-1.10	0.391
>32 weeks	0.97	0.80-1.18	0.768	0.92	0.73-1.16	0.466
*Delay from screening to ART initiation*						
<14 days	1.0	(ref)		1.0	(ref)	
14-20 days	0.95	0.80-1.12	0.549	0.96	0.81-1.14	0.655
21-27 days	0.97	0.80-1.18	0.777	0.95	0.77-1.17	0.654
28-34 days	1.07	0.90-1.26	0.448	1.04	0.85-1.26	0.708
>35 days	0.89	0.72-1.03	0.264	0.86	0.67-1.09	0.206

Associations are shown as risk ratios (RR) with 95% confidence intervals (CI).

* Adjusted associations are adjusted for all covariates shown.

## Discussion

This analysis shows that pregnant women who are referred to an adult ART service for treatment initiation may face significant delays in initiating ART. As a result of these delays, a sizable proportion of women in this setting did not initiate ART before delivery. Among women who did initiate ART during the antenatal period, there was substantial variability in the delays from women’s first screening at the ART clinic to starting therapy, but the duration of systemic delays before ART initiation was not associated with improved patient outcomes on treatment. Given the urgency of ART initiation in pregnancy to prevent vertical HIV transmission, these data have important implications for the design of ART services for eligible pregnant women.

The median delay from screening at the ART clinic to ART initiation was 21 days (IQR, 14–29). When coupled with a median gestation at screening of 27 weeks, most women received 8 weeks or less of therapy prior to delivery. While the duration of ART required in pregnancy to achieve viral suppression is influenced by pre-ART viraemia and the antiretroviral drugs used, most studies show that at least 12–20 weeks of therapy is required to achieve viral suppression in most women and in turn minimize the risk of vertical HIV transmission [9,21]. Following from this, while we did not measure viral suppression at delivery or infant HIV outcomes as part of this routine service, it is likely that a proportion of the women had significant viraemia and, therefore, were at increased risk of HIV transmission at the time of delivery [21].

Here we analyzed the delay from screening to initiation of ART as a proxy for the time available for patient preparation before starting therapy. This delay may influence patient preparation in two related ways. First, increased time before starting ART provides time for formal patient counseling sessions, as group counseling sessions take place at regularly scheduled intervals each week at the ART clinic. Second, it is possible that some period of time before ART initiation may be useful to help some patients adjust individually to the need to start lifelong therapy, as ART initiation is accompanied by psychosocial stressors related to HIV stigma and disclosure, and may be particularly complex in the context of pregnancy [22]. It is important to note that the delay variable used here cannot distinguish these two constructs, and we do not have measures of the number of counseling sessions attended nor any markers of patients’ perceived readiness to initiate therapy. Nonetheless, the overall delay between screening and ART initiation is a useful indirect measure of both phenomena in combination.

Although delays from ART screening to initiation limited the total duration of ART received before delivery, these data suggest that there was no apparent benefit to these delays on maternal outcomes. Levels of retention in care appeared relatively low but viral suppression was relatively high among those retained. However neither of these outcomes varied according to the length of delay from screening to ART initiation. This finding is in keeping with the general literature on patient preparation before ART initiation: while there are hypotheses that delaying ART initiation for patient preparation contributes to improved outcomes [15,16], there are few empirical data that support these hypotheses [18,23]. In the absence of such an association, the benefits of delaying ART initiation in pregnancy for adherence preparation and counseling are unclear.

This is one of the first studies to investigate the impact of systemic delays before ART initiation during pregnancy on ART outcomes. There are other populations of HIV-infected individuals in which systemic delays to ART initiation contribute to avoidable morbidity and mortality, including adults with advanced immunosuppression [24], individuals with tuberculosis [25], and perinatally-infected infants [26] (although there are other clinical situations in which rapid ART initiation may lead to clinical deterioration [27]). In each of these groups, observational evidence followed by definitive randomized controlled trials has shown that the risks associated with delayed ART initiation are outweighed by the benefits of expedited therapy. Timing of ART initiation in pregnancy is quite different from these examples, however: HIV-infected pregnant women are usually relatively healthy; the short-term benefits of rapid ART initiation are primarily in reducing transmission risk; and the principle threats to the retention of pregnant women on ART may be loss to follow-up and inadequate adherence rather than morbidity and mortality. In this light, while these results could be interpreted to support a ‘test-and-treat’ approach to ART in pregnancy [28,29] - where all HIV-infected pregnant women are initiated on ART immediately, regardless of clinical or immunological status – these findings should be interpreted with care. In particular, the data come a group of women with relatively low CD4 cell counts (median, 142 cells/μL) attending a single, relatively large ART clinic in South Africa. The results may not be generalizable to other geographic settings and populations of pregnant women, and there is a clear need for additional investigation of any ‘test-and-treat’ approach to manage HIV in pregnancy.

The findings reported here should be viewed in light of several important limitations. Because information was taken from a retrospective review of patient records, we do not have available several measures of interest, in particular data on deliveries, neonatal health, and infant HIV testing outcomes. In addition, women commonly arrived at the ART clinic having taken zidovudine PMTCT prophylaxis for several weeks, clouding the interpretation of ‘baseline’ pre-ART viral loads. After ART initiation, viral load testing was conducted according to women’s duration on ART and there was not viral load testing at the time of delivery; thus we have little insight into the overall proportion of women who achieved viral suppression by delivery. Moreover we do not have detailed information on the precise reasons why ART initiation was expedited in some women and delayed in others: if the reasons underlying the length of this delay are associated with women’s outcomes on ART, this may represent an important, unmeasured confounding factor. Such confounding by indication is commonplace throughout clinical research [30], including studies of the timing of ART initiation [31], and can only be comprehensively addressed through randomized studies [32].

Despite these limitations, this study has important implications for services to initiate ART in eligible pregnant women. There are well-established risks associated with delaying ART initiation in pregnancy, in terms of vertical transmission of HIV [10] as well as maternal morbidity and mortality [33]. While a potential benefit associated with delayed ART initiation has been suggested in terms of improved maternal outcomes on treatment (due to increased time for patient preparation), these data suggest no such benefit. In turn, the need to expedite ART in pregnancy is likely to outweigh the putative benefits of delaying therapy for patient education. Although patient education and counseling is a critical part of ART initiation, the usefulness of delaying ART until after patient education and counseling is complete is unclear. This points in turn to the need for strategies to initiate ART as quickly as possible in the context of pregnancy, but few models for this exist, and any intervention strategy must address the multiple factors that combine to delay ART initiation in pregnancy [34].

## Conclusions

In summary, these data show that that ART-eligible pregnant women experience significant delays before beginning treatment, and that such delays are not associated with improved maternal health outcomes. Given the need to maximize the duration of ART before delivery, there is an urgent need for new strategies to help expedite ART initiation in pregnancy to promote both maternal and child health.

## Competing interests

The authors declare that they have no competing interests.

## Authors’ contributions

LM, LGB and EJA designed the study; RZ collected and analysed the data; LM and RZ wrote the first draft of the manuscript; LGB and EJA made substantial contributions to the manuscript. All authors read and approved the final manuscript.

## Pre-publication history

The pre-publication history for this paper can be accessed here:

http://www.biomedcentral.com/1471-2393/12/94/prepub
